# The accuracy of a novel pedicle screw insertion technique assisted by a special angular scale in the subaxial cervical spine using lateral mass as a reference marker

**DOI:** 10.1186/s13018-020-02054-1

**Published:** 2020-11-23

**Authors:** Hang Shi, Lei Zhu, Jun Ma, Yu-Cheng Zhu, Xiao-Tao Wu

**Affiliations:** 1 Department of Spine Surgery, Zhongda Hospital, School of Medicine, Southeast University, Nanjing, 210009 Jiangsu China; 2 The Affiliated Suqian Hospital of Xuzhou Medical University, Suqian, China

**Keywords:** CPS, Special angular scale, Lateral mass, Optimal entry point, PTA, PLMA

## Abstract

**Background:**

Posterior cervical pedicle screw (CPS) internal fixation has better biomechanical stability than other posterior cervical fixation methods. However, this technique is limited in clinical practice due to the complex anatomical structure and the adjacent relationship of the cervical pedicle, and the high risk of neurovascular injury. The purpose of this study was to describe a novel subaxial CPS insertion technique assisted by a special angular scale using lateral mass as a reference marker and to evaluate the accuracy of CPS placement and the distribution characteristics of CPS misplacement.

**Methods:**

A total of 36 patients with subaxial cervical spine diseases who underwent posterior CPS fixation were consecutively selected. The optimal entry point on the posterior surface of the lateral mass was identified on the three-dimensional cervical model reconstructed from preoperative computed tomography (CT) images. The pedicle transverse angle (PTA) and pedicle-lateral mass angle (PLMA) were measured on the transverse and sagittal CT images respectively. The pedicle screws were inserted according to the preoperatively planned entry point and angles. We analysed the postoperative CT images for CPS misplacement rates and perforation directions following the Lee classification.

**Results:**

Overall, 177 pedicle screws were inserted, of which 119 (67.2%) were classified as grade 0, 43 (24.3%) as grade 1, 12 (6.8%) as grade 2 and 3 (1.7%) as grade 3 by the postoperative CT images. The accuracy rate of CPS placement was 91.5%. Of the 15 misplaced pedicle screws (grades 2 and 3), 11 were lateral pedicle perforations, 3 were superior perforations and 1 was an inferior perforation. There were no neurovascular injuries related to CPS misplacement.

**Conclusions:**

With our technique, the optimal entry point and two angles (PTA and PLMA) were identified for CPS insertion. The novel CPS insertion technique assisted by a special angular scale provides high accuracy and few complications.

## Introduction

With the development of cervical surgery techniques, posterior cervical internal fixation has been widely applied in the treatment of various subaxial cervical spine diseases. The commonly used subaxial cervical posterior screw fixation methods include pedicle screw, lateral mass screw, lamina screw and transfacet screw [1–3]. Many studies have reported that the cervical pedicle screw (CPS) method has better biomechanical stability than other posterior cervical fixation methods [1, 4]. Nevertheless, there are potential risks of injury to the surrounding neurovascular tissues, such as the spinal cord, vertebral arteries and nerve roots, as a result of CPS misplacement due to the anatomical variability of the cervical pedicle, a small pedicle size or a large pedicle transverse angle (PTA) [5–7].

A variety of CPS insertion strategies have been advocated to improve the accuracy of screw placement and avoid neurovascular injury complications. Some innovative techniques, such as navigation systems, are restricted because of the high cost and the added procedure time [8]. Therefore, freehand CPS insertion techniques are still widely used in clinical practice [9]. In this study, we describe a novel freehand CPS insertion technique assisted by a special angular scale using lateral mass as a reference marker and evaluate the accuracy of CPS placement and the distribution characteristics of CPS misplacement in patients with various subaxial cervical spine diseases.

## Materials and methods

### Patient population

From January 2014 to December 2018, 36 patients with subaxial cervical spine diseases who underwent the posterior cervical pedicle fixation using the novel CPS insertion technique were consecutively selected. There were 23 males and 13 females with an average age of 53 years (range 27–75 years). The diagnoses included trauma (6 patients), cervical spondylotic myelopathy and spinal stenosis (15 patients), ossification of the posterior longitudinal ligament (9 patients) and spinal cord tumour (6 patients). The study was performed in compliance with ethical standards and was approved by the institutional review board of our hospital. The inclusion criteria were patients aged at least 18 years, no previous cervical spine surgery and clear images from the third through seventh cervical vertebrae. Patients with infectious, cervical vertebral pedicle damage, cervical vertebral rotation, congenital cervical deformity or anatomical variation were excluded from the study. The follow-up methods involved outpatient service, e-mail and telephone call surveys to assess postoperative recovery and determine whether any neurovascular injury or other complications related to CPS misplacement developed.

### Preoperative examination

All patients underwent preoperative radiography, computed tomography (CT) and magnetic resonance imaging (MRI) scans. Multiplanar reconstruction of the target cervical vertebra was performed by the transverse CT images.

**Fig. 1:**
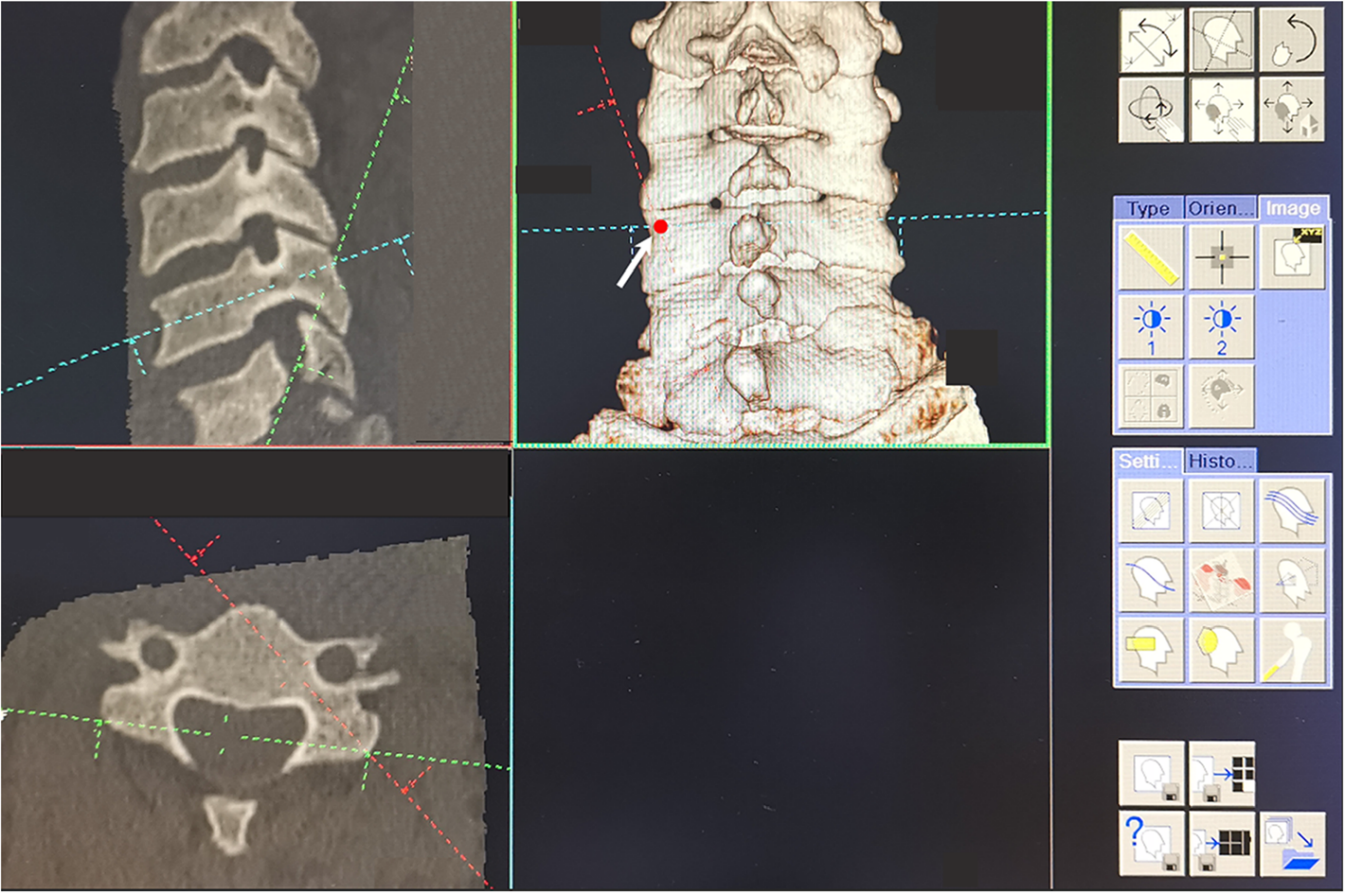
The ideal pedicle trajectory was defined as a line passing through the centre of the pedicle on the transverse and sagittal CT images (left column). The three-dimensional cervical model was reconstructed using cervical spine transverse CT images. On the three-dimensional reconstructed cervical model, the ideal pedicle trajectory would penetrate the posterior surface of the lateral mass, and this point was then identified as the optimal entry point passing through the centre of the pedicle (right column)

**Fig. 2:**
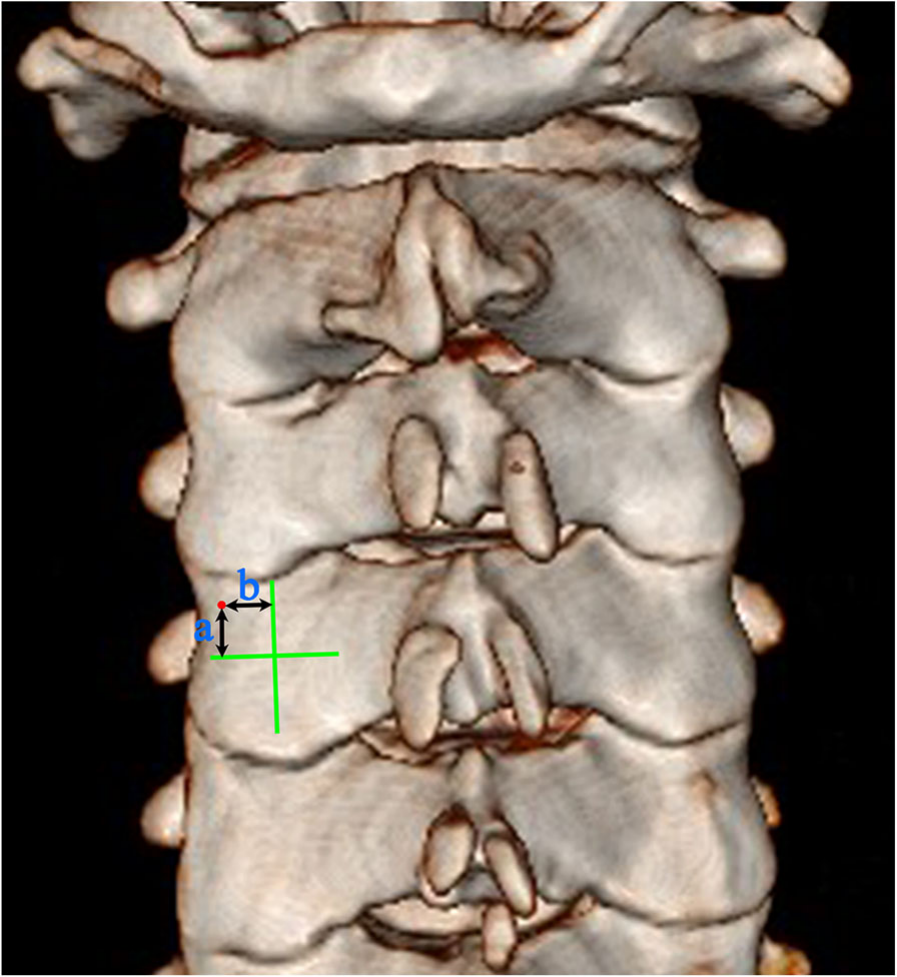
Vertical and horizontal offsets of the optimal entry point (**a**, **b**) were measured from the centre of the lateral mass

**Fig. 3:**
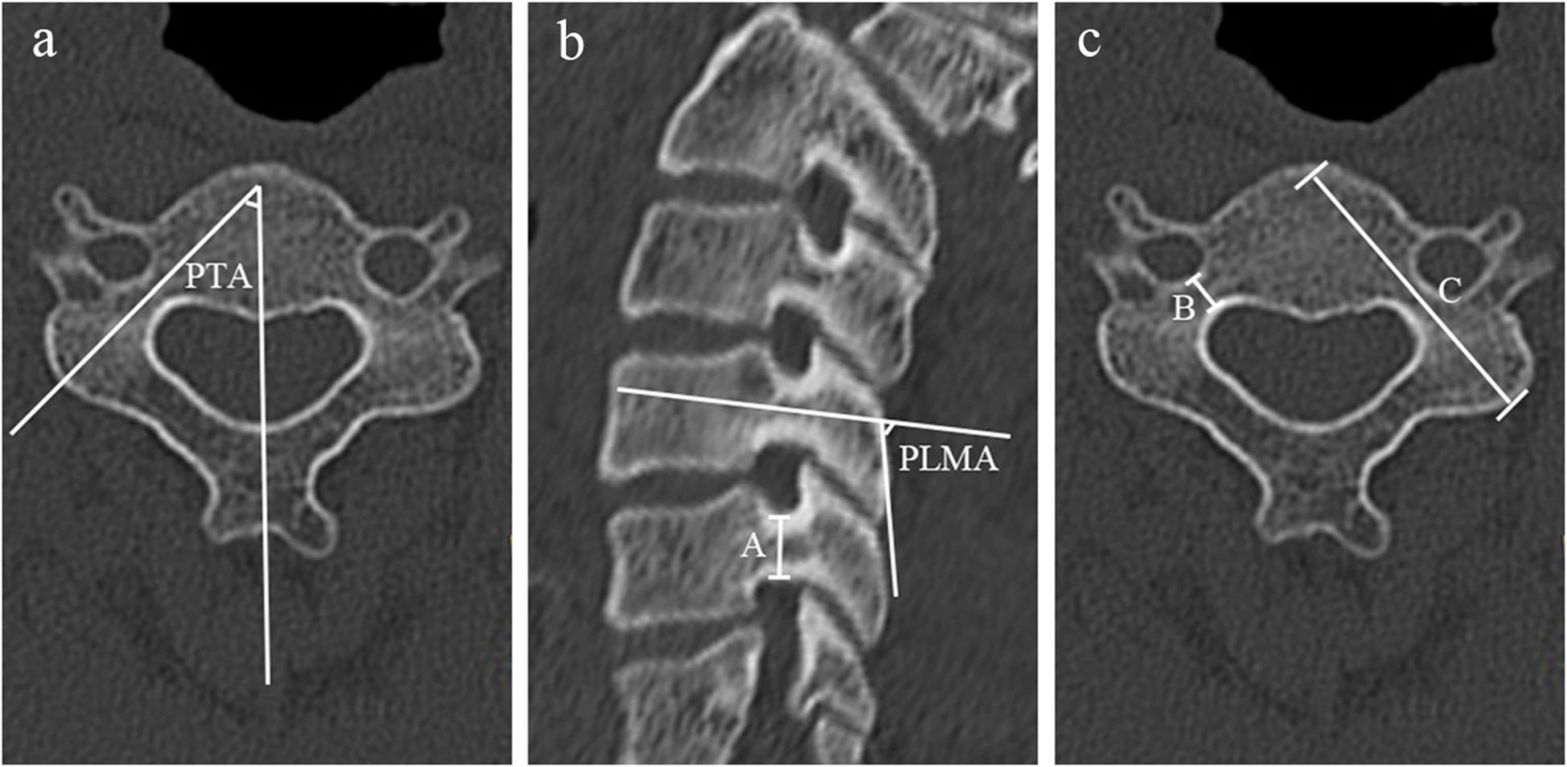
The preoperative pedicle transverse angle (PTA) and pedicle-lateral mass angle (PLMA) were measured on the transverse and sagittal CT images, respectively (**a**, **b**), and other parameters, including the pedicle height (A), pedicle width (B) and pedicle axis length (C), were also measured (**b**, **c**). The PTA referred to the included angle between the pedicle trajectory and the vertical line of the posterior edge of the vertebral body on the transverse CT images, and the PLMA was defined as the included angle between the pedicle trajectory and the posterior edge of lateral mass on the sagittal CT images

**Fig. 4:**
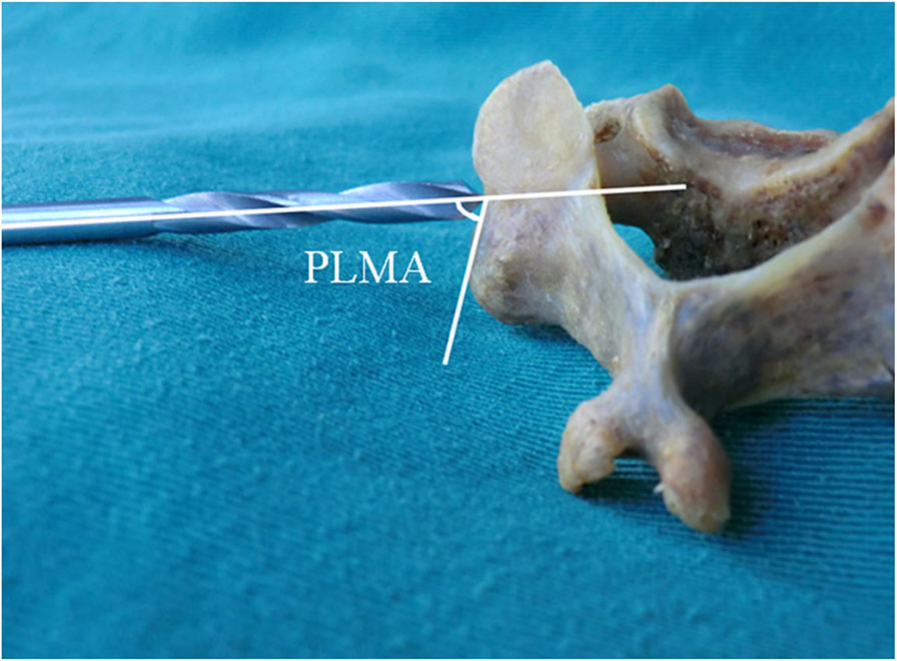
The diagram of the pedicle-lateral mass angle (PLMA) on a specimen

The ideal pedicle trajectory was defined as a line passing through the centre of the pedicle on the transverse and sagittal CT images. The three-dimensional cervical model was reconstructed using cervical spine transverse CT images. On the three-dimensional reconstructed cervical model, the ideal pedicle trajectory would penetrate the posterior surface of the lateral mass, and this point was then identified as the optimal entry point passing through the centre of the pedicle (Fig. 1). Vertical and horizontal offsets of the optimal entry point were measured from the centre of the lateral mass (Fig. 2). The preoperative PTA and pedicle-lateral mass angle (PLMA) were measured on the transverse and sagittal CT images, respectively, and other parameters, including the pedicle width, pedicle height and pedicle axis length, were also measured (Fig. 3). The PTA referred to the included angle between the pedicle trajectory and the vertical line of the posterior edge of the vertebral body on the transverse CT images, and the PLMA was defined as the included angle between the pedicle trajectory and the posterior edge of lateral mass on the sagittal CT images. The PLMA on the specimen was shown in Fig. 4.

### Surgical procedure

**Fig. 5:**
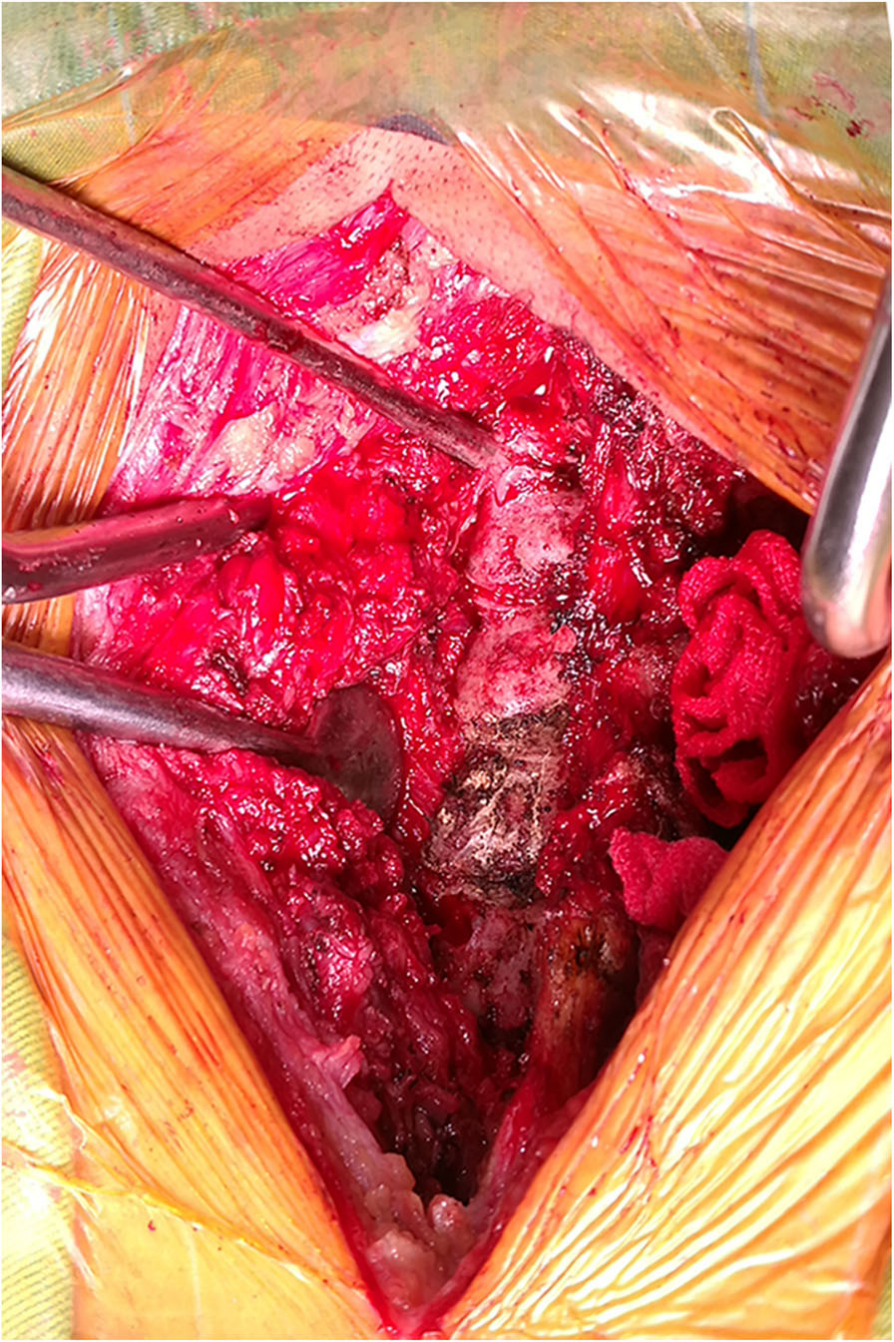
The lateral masses of target segments were clearly exposed during operation

**Fig. 6:**
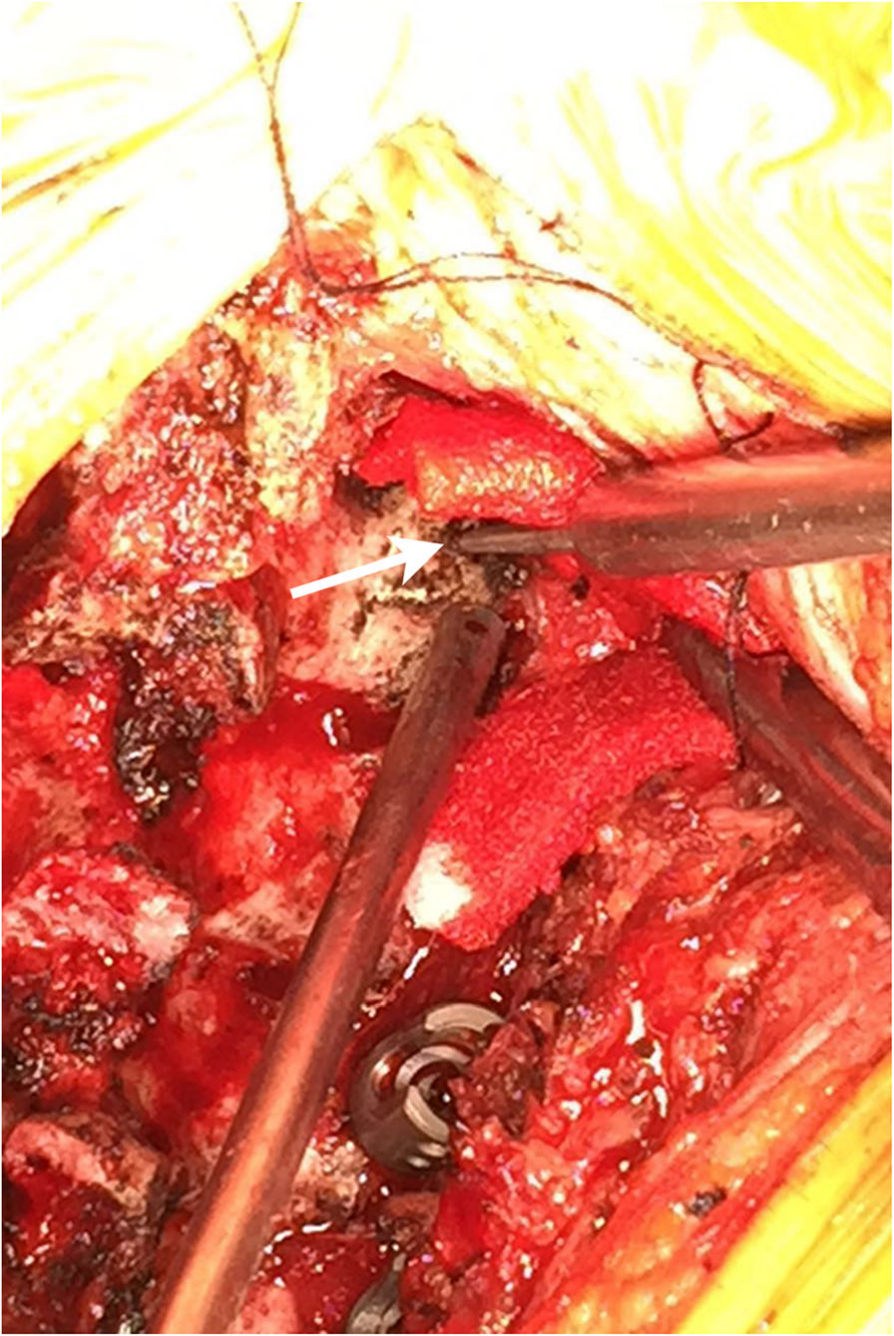
The entry hole was made by using a power drill at the optimal entry point on the posterior surface of the lateral mass

**Fig. 7:**
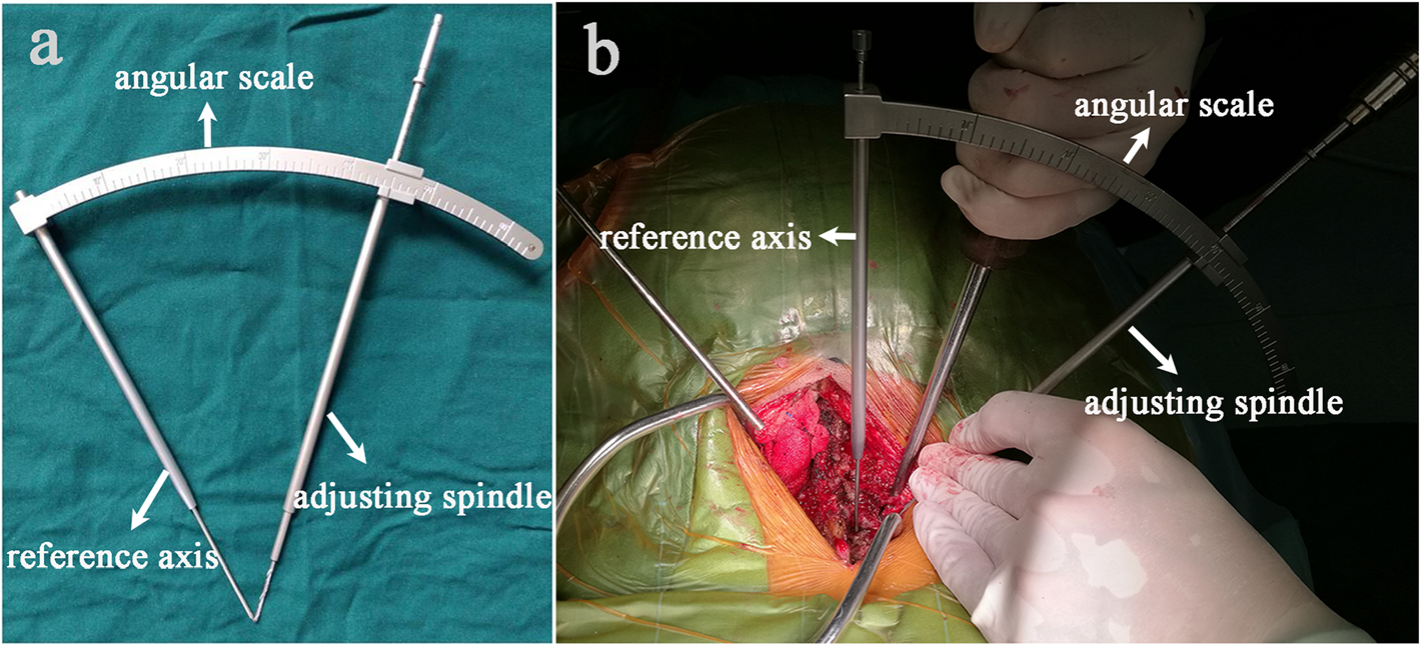
A special angular scale that controls the angle (**a**). The pedicle screw was inserted according to preoperative measurements of pedicle transverse angle (PTA) and pedicle-lateral mass angle (PLMA) (**b**). The detailed processes were as follows: during the operation, the reference axis was placed parallel to the spinous processes of the cervical spine, and then the adjusting spindle was regulated to achieve the preset PTA. To control PLMA as accurately as possible in clinical practice, after identifying the entry hole, the wire tapping inside the adjusting spindle was first placed perpendicular to the lateral mass and then slightly adjusted to the head or tail side according to the preset PLMA

**Fig. 8:**
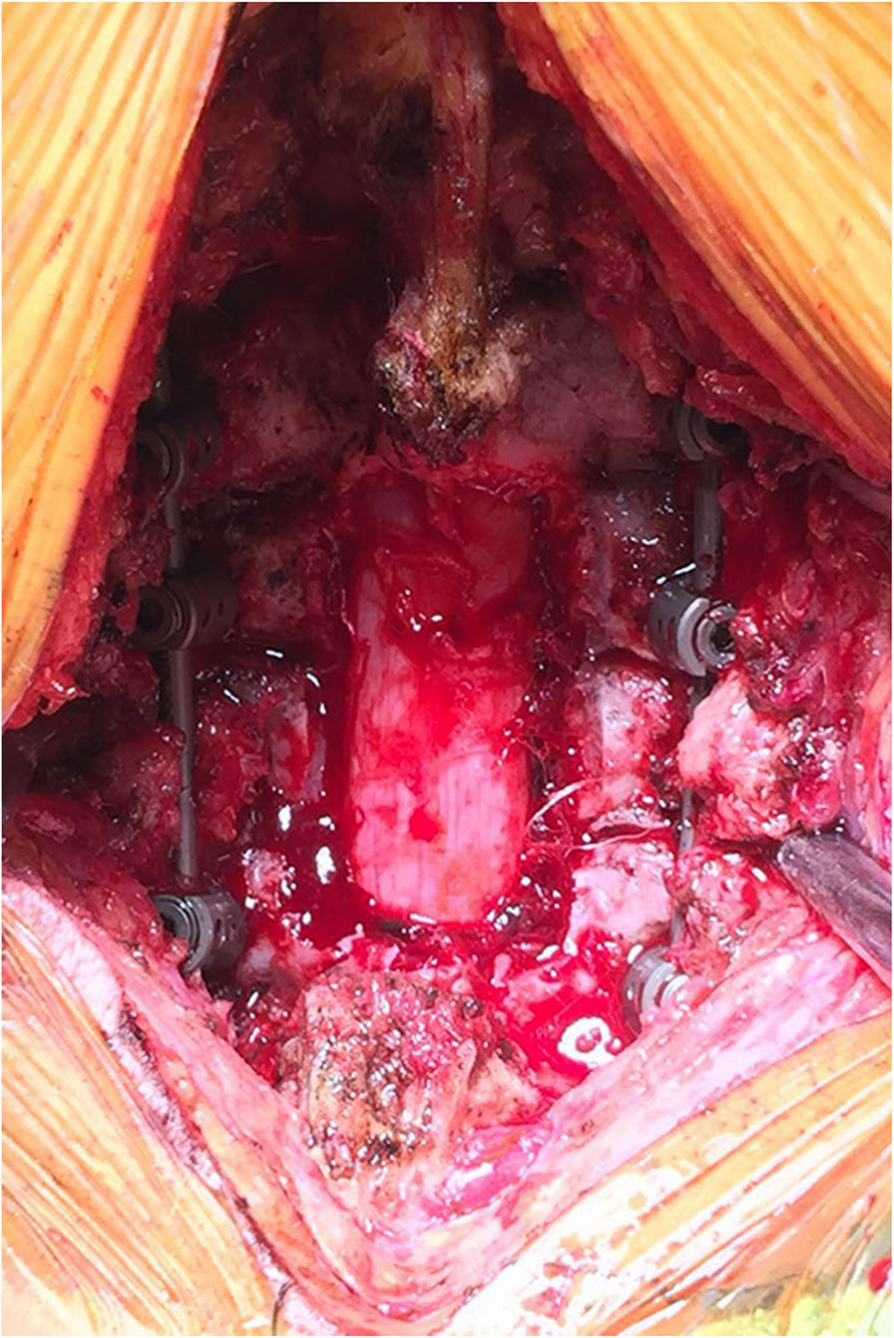
The rod with the appropriate size was bent in accordance with normal cervical curvature and the connections between the screws and rod were tightened

All patients were placed in the prone position with their skull fixed using a Mayfield clamp. Ensure that the cervical spine process was in the middle and there was no lateral tilt. A straight posterior midline incision was made. The cervical lamina and lateral mass were fully exposed by dissecting the paravertebral muscles during the operation (Fig. 5). The centre of the lateral mass was marked, and the optimal entry point on the posterior surface of the lateral mass was then identified following the preoperative measurements of the vertical and horizontal offsets. An entry hole was made by using a power drill at the optimal entry point (Fig. 6) and the pedicle canal was created with a special angular scale combined with a wire tapping according to the preoperative measurements of the PTA and PLMA (Fig. 7). The detailed processes were as follows: during the operation, the reference axis was placed parallel to the spinous processes of the cervical spine, and then the adjusting spindle was regulated to achieve the preset PTA. To control PLMA as accurately as possible in clinical practice, after identifying the entry hole, the wire tapping inside the adjusting spindle was first placed perpendicular to the lateral mass and then slightly adjusted to the head or tail side according to the preset PLMA. The pedicle canal was ascertained with a probe to ensure its safety, and then, a pedicle screw with a diameter of 3.5 mm and a length of 20–24 mm was inserted. Laminectomy, laminoplasty or tumour removal were performed for posterior decompression. Finally, the rod with the appropriate size was bent in accordance with normal cervical curvature and the connections between the screws and rod were tightened (Fig. 8).

### Postoperative imaging evaluation of CPS placement

A postoperative CT scan and multiplanar reconstruction of the fixed segments were performed, and the CT images were assessed by an independent radiologist and an experienced spine surgeon who did not perform the surgery and was blind to the surgery. The accuracy of CPS placement was evaluated according to the Lee classification [10]: grade 0, no perforation; grade 1, perforation less than 25% of the screw diameter; grade 2, perforation from 25% to 50% of the screw diameter; and grade 3, perforation more than 50% of the screw diameter. CPSs with grades 0 and 1 were regarded as accurately placed screws, and those with grades 2 and 3 were regarded as misplaced screws. The directions of pedicle perforation were classified into four categories: inferior, superior, medial and lateral.

### Postoperative complications analysis

Postoperative complications such as spinal cord injury, vertebral artery injury, nerve root injury or internal fixation failure resulting from CPS misplacement were recorded and analysed.

### Statistical analysis

All data were analysed using SPSS Statistics software (version 22.0, SPSS Inc, Chicago, IL, USA). Continuous variables were summarised using mean ± standard deviation (SD). The differences in the preoperative angles of two sides were analysed using *t* test. The differences in the CPS misplacement rates and perforation directions were analysed using Fisher’s exact test, with *P* < 0.05 considered to be statistically significant.

## Results

**Fig. 9:**
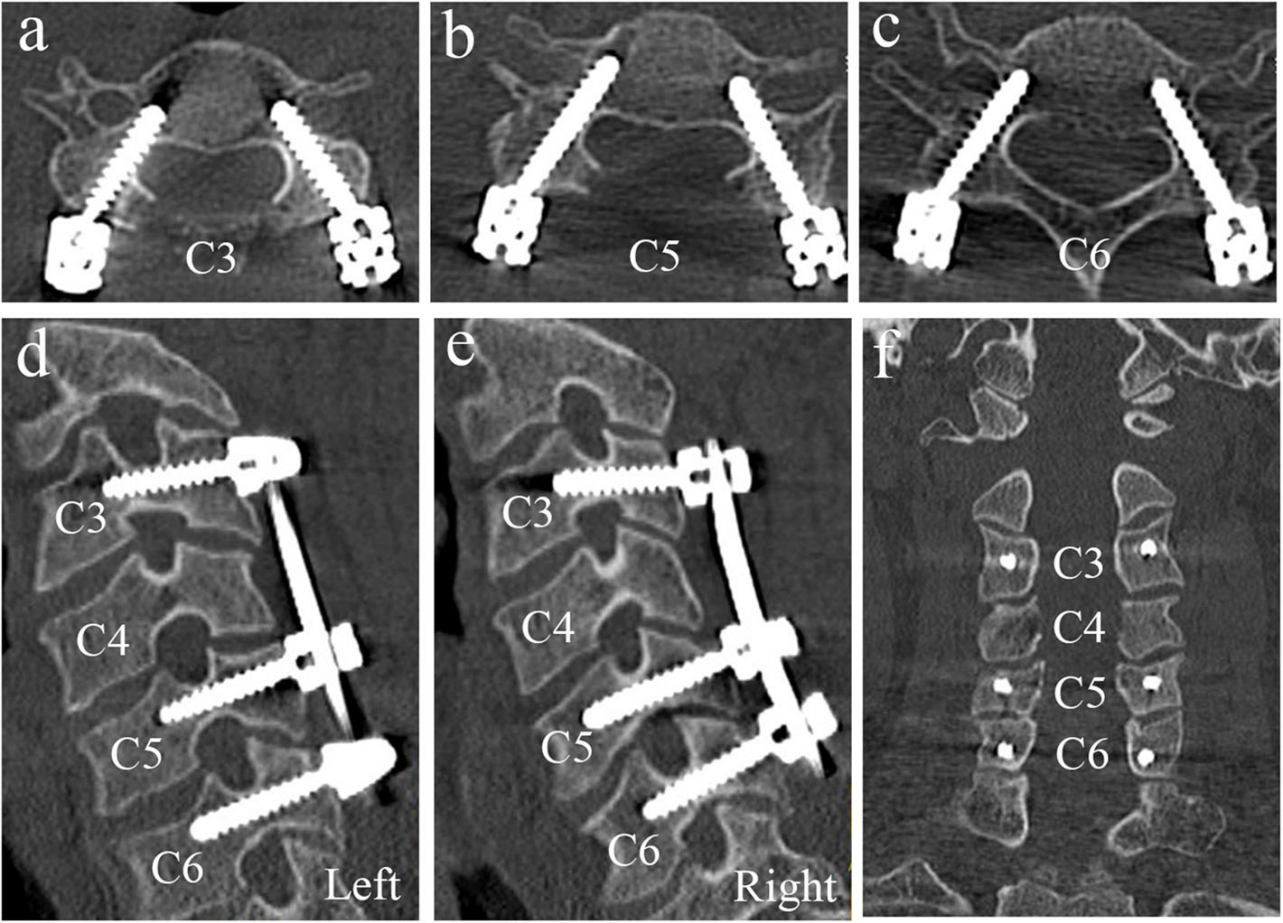
A typical case with successful cervical pedicle screw (CPS) fixation. Postoperative CT scans and multiplanar reconstruction showed the good placement of pedicle screws at C3, C5, C6 levels (**a**, **b**, **c**, **d**, **e**, **f**)

All patients underwent the surgeries successfully. There were 5 incidences of conversion to lateral mass screws due to muscle obstruction. A total of 177 pedicle screws were inserted, including 90 on the left and 87 on the right. A total of 112 and 65 pedicle screws were inserted into male and female patients, respectively. The measurements of preoperative PTA and PLMA are shown in Table 1. There was no significant difference between the two sides of each pedicle (*P* > 0.05). The PTA has a decreasing trend from C3 to C7, ranging from 25° to 51°. However, the PLMA has a trend of increasing from C3 to C7, ranging from 68° to 103°. The segmental distribution and classification of all pedicle screws are shown in Table 2. Of 177 pedicle screws, 119 (67.2%) were classified as grade 0 (no perforation), 43 (24.3%) as grade 1 (perforation less than 25% of the screw diameter), 12 (6.8%) as grade 2 (perforation between 25 and 50% of the screw diameter) and 3 (1.7%) as grade 3 (perforation more than 50% of the screw diameter). The accuracy rate of CPS placement was 91.5% (162/177) (Fig. 9) and the misplacement rate was 8.5% (15/177).

**Table 1:** Measurements of the Preoperative PTA and PLMA

Pedicle	PTA (°)		PLMA (°)	
	Left	Right	Left	Right
C3	44.8 ± 3.1 (40–50)	43.9 ± 2.8 (39–49)	77.8 ± 4.3 (72–86)	77.5 ± 4.5 (68–85)
C4	43.2 ± 3.4 (37–48)	43.5 ± 3.6 (39–51)	79.1 ± 3.7 (74–89)	79.6 ± 6.0 (72–93)
C5	42.3 ± 4.0 (33–51)	42.6 ± 3.1 (38–49)	83.3 ± 4.4 (76–94)	82.8 ± 4.6 (78–96)
C6	38.7 ± 4.4 (31–45)	38.7 ± 4.3 (29–47)	87.6 ± 6.2 (78–101)	86.7 ± 4.7 (82–102)
C7	33.9 ± 3.7 (25–42)	35.2 ± 2.7 (29–40)	90.3 ± 5.3 (80–102)	89.7 ± 5.2 (83–103)

Data are presented as mean ± standard deviation.

*PTA* pedicle transverse angle, *PLMA* pedicle-lateral mass angle

**Table 2:** Segmental distribution and classification of 177 pedicle screws in 36 patients

Grade	C3	C4	C5	C6	C7
0	12	19	24	35	29
1	7	8	13	9	6
2	3	4	3	1	1
3	1	1	1	0	0
Total	23	32	41	45	36

The perforation directions of the misplaced pedicle screws are shown in Table 3. Of the 15 misplaced pedicle screws (grades 2 and 3), 11 were lateral wall perforations, 3 were superior wall perforations, 1 was an inferior wall perforation and there were no medial wall perforations. The screws with perforations of the lateral wall accounted for 73.3% (11/15) of all the misplaced screws, and this rate was higher than that of the superior wall (20%, 3/15), the inferior wall (6.7%, 1/15) and the medial wall (0, 0/15). There were 9 and 6 misplaced screws on the left and right sides, respectively. There was no significant difference between the two sides (*X*^2^ = 0.55, *P* = 0.46). There were 11 and 4 misplaced screws in male and female patients, respectively. There was no significant difference between female and male patients (*X*^2^ = 0.71, *P* = 0.40).

**Table 3:** Perforation directions of 15 misplaced pedicle screws

Pedicle	Lateral*	Superior	Inferior	Medial
C3	4	0	1	0
C4	2	1	0	0
C5	3	1	0	0
C6	1	0	0	0
C7	1	1	0	0
Total	11	3	1	0

*Statistically significant using chi-squared tests *P* < 0.05

One patient with cervical trauma and quadriplegia died of pulmonary infection and respiratory failure 1 month after the operation and was lost during follow-up. The other patients were followed up for 12–28 months (mean of 18 months). None of the patients had neurovascular injuries related to CPS misplacement.

## Discussion

Posterior CPS internal fixation has the advantages of strong pull-out resistance and excellent biomechanical stability in the treatment of cervical fractures, tumours, ossification of posterior longitudinal ligament and other cervical spine diseases [11]. However, this technique is limited in clinical practice due to the complex anatomical structure and the adjacent relationship of the cervical pedicle, and the high risk of vascular, nerve root and spinal cord injury. Studies have shown that the incidence of complications related to screw misplacement is high, and some of these complications can result in serious consequences [12, 13]. At present, the technology commonly used for CPS placement mainly includes freehand screw placement technology, computer-assisted navigation technology and 3D-guided template technology. The latter two technologies can improve the safety and accuracy of CPS placement [14, 15]. However, computer-assisted navigation technology has the disadvantages of requiring expensive equipment, a long operative time and a high radiation dose [16]. 3D-guided template technology requires complex preoperative designs, so it is not suitable for emergency surgery patients. Therefore, freehand screw placement technology is still widely applied in clinical practice.

Many former surgeons [17, 18] measured the included angle between the upper endplate of the vertebral body and the central axis of the pedicle on the sagittal CT images before surgery and used this measurement for the sagittal plane angle for pedicle screw placement. Then the pedicle screw was placed in accordance with the preoperative measurement angle. Patients commonly underwent preoperative CT scans with the cervical vertebra in the neutral position. However, patients were placed in the prone position during the operation. The cervical curvature cannot reach an agreement between preoperative and intraoperative position, especially for patients with cervical instability or deformity. The preoperative and intraoperative sagittal angle error may increase the risk of screw misplacement and related complications. The intraoperative insertion angle during the process of CPS insertion should be the same as that measured before the operation. The ideal reference plane and angle of screw insertion should not change with the patient position. Therefore, the posterior surface of the lateral mass, which was a visible and constant reference, was used as a reference marker for the sagittal plane angle for CPS insertion. Then, the PLMA was measured, which did not change with the changes in cervical flexion and extension. In the study, the PTA and PLMA were measured on the transverse and sagittal CT images, respectively. The optimal entry point on the posterior surface of the lateral mass was also identified on the three-dimensional cervical model reconstructed before surgery. The CPS was placed with the same reference marker, entry point and angles during the operation. The accuracy rate of CPS placement was 91.5% (162/177), which was higher than the accuracy (84.9%) reported by Lee et al. [10]. The results of our research showed that the misplacement rate of CPS placement can be reduced by the novel pedicle screw insertion technique assisted by a special angular scale using lateral mass as a reference marker.

The directions of pedicle perforation were classified into four categories: inferior, superior, medial and lateral. In this study, screws with perforations of the pedicle lateral wall accounted for 73.3% (11/15) of all the misplaced screws, and this rate was higher than those of the other three walls; this result is consistent with the results reported by Park et al. [6]. In addition to the fact that the bony cortex of the pedicle lateral wall is thin, we believe that the following factors are also the reasons that the screw penetrated the pedicle lateral wall easily. First, the PTA of the subaxial cervical spine is large, measuring up to 52° [19]. Due to the obstruction of soft tissues, such as paravertebral muscles, the inclination angle of the screw cannot be sufficiently large during CPS insertion, especially for C3–C5 pedicle screw placement [20]. In addition, for some patients with cervical spine injury or cervical instability, although there is no obvious rotation of the cervical vertebra in the preoperative CT scans, the cervical vertebra may rotate toward the opposite side due to the stress of drilling during the screw placement process, which is also one of the reasons for the perforation of the lateral wall. Sugimoto et al. [21] reported that 76 pedicle screws were placed in 17 patients with cervical spine injury, and the vertebral rotation was measured during screw insertion. The average degree of cervical vertebral rotation was 9.1° at C3, 7.8° at C4, 6.7° at C5, 4.9° at C6 and 2.8° at C7. In our study, the assistant used a forcep to temporarily immobilise the spinous process of the cervical spine during the screw placement process to avoid cervical vertebral rotation. Besides, the screws with perforations of the superior and inferior wall accounted for 26.7% (4/15) of all the misplaced screws in our study. This may be due to an error between the actual PLMA and the preset PLMA during the operation, which further resulted in the tilt of the pedicle screws to the cephalic or caudal sides.

Not all misplaced screws cause complications. In a study reported by Nakashima et al. [12], 390 cervical pedicle screws were inserted into 84 patients and 76 of these pedicle screws were misplaced. The rate of misplacement was 19.5%. Complications related to screw misplacement occurred in 5 patients, including 3 patients with nerve root injury and 2 patients with vertebral artery injury. In a study described by Yoshihara et al. [22], 2668 subaxial cervical pedicle screws were inserted into 661 patients. The incidence of vertebral artery injury was 0.61% (4/661), and the incidence of nerve root injury was 0.31% (2/661). In our study, a total of 177 pedicle screws were inserted into 36 patients, and 15 of these pedicle screws were misplaced. However, there were no neurovascular injuries related to screw misplacement.

Although the screw placement technique proposed in this study has high accuracy and few complications, it also has some limitations. First, the key to the technique is to determine one optimal entry point and two angles (PTA and PLMA). The PTA is defined as the included angle between the central axis of the pedicle and the vertical line of the posterior edge of the vertebral body on the transverse CT images. However, for some patients with a cervical rotatory deformity or articular interlocking caused by cervical spine injury, the vertical line of the posterior edge of the vertebral body also rotates synchronously, which leads to preoperative and intraoperative PTA errors and increases the risk of the pedicle screw penetrating the medial or lateral wall. Second, for some patients with a cervical deformity or an anatomical structure variation of the lateral mass, the included angle between the central axis of the pedicle and the posterior surface of the lateral mass cannot be accurately measured, and the optimal entry point on the posterior surface of the lateral mass cannot be precisely identified. Third, in this study, PTA can be precisely controlled by the special angular scale, but PLMA cannot be precisely quantified. We can find that the PLMA is getting closer and closer to 90° from C3 to C7 (Table 1). To control PLMA as accurately as possible in the clinical practice of this study, after identifying the entry hole, the wire tapping inside the adjusting spindle was first placed perpendicular to the lateral mass and then slightly adjusted to the head or tail side according to preoperative measurement PLMA. Furthermore, compared with the research results of other scholars, there are some errors in the accuracy of screw placement because there is no uniform standard for screw misplacement.

## Conclusions

This study shows results for a novel CPS insertion technique assisted by a special angular scale using the lateral mass as a reference marker. With our technique, the optimal entry point and two angles (PTA and PLMA) are identified before the operation, and the screws are inserted with the same entry point and angles during the operation. The novel CPS insertion technique provides high accuracy and few complications.

## Supplementary Information

**Additional file 1.**

## Data Availability

All data generated or analysed during this study are included in its supplementary information files.

## Abbreviations

CPS: Cervical pedicle screw
CT: Computed tomography
PTA: Pedicle transverse angle
PLMA: Pedicle-lateral mass angle
MRI: Magnetic resonance imaging
