# Supplementary material for: The accuracy of a novel pedicle screw insertion technique assisted by a special angular scale in the subaxial cervical spine using lateral mass as a reference marker
Source: J Orthop Surg Res. 2020 Nov 23;15:551. doi: 10.1186/s13018-020-02054-1 (PMC7681976; doi:10.1186/s13018-020-02054-1)
Supplement: Supplementary file 1 — Additional file 1. [file 13018_2020_2054_MOESM1_ESM.doc]

C3 (12 cases)

| case | gender | age | PTA (°) | | PLMA (°) | |
| --- | --- | --- | --- | --- | --- | --- |
|  |  |  | C3R | C3L | C3R | C3L |
| 1 | M | 48 | 46 | 46 | 72 | 77 |
| 2 | M | 50 | 42 | 44 | 86 | 80 |
| 3 | F | 57 | 43 | 45 | 81 | 85 |
| 4 | M | 40 | 44 | 47 | 82 | 74 |
| 5 | M | 42 | 50 | 45 | 77 | 79 |
| 6 | M | 44 | 45 | 42 | 74 | 68 |
| 7 | F | 61 | 40 | 43 | 73 | 72 |
| 8 | M | 49 | 44 | 39 | 80 | 78 |
| 9 | F | 48 | 42 | 43 | 79 | 81 |
| 10 | F | 61 | 44 | 40 | 74 | 76 |
| 11 | M | 52 | 50 | 44 | 75 | 79 |
| 12 | M | 64 | 47 | 49 | 80 | 81 |

| Descriptive Statistics | | | | | |
| --- | --- | --- | --- | --- | --- |
|  | N | Minimum | Maximum | Mean | Std. Deviation |
| VAR00001 | 12 | 40.00 | 50.00 | 44.7500 | 3.07852 |
| VAR00002 | 12 | 39.00 | 49.00 | 43.9167 | 2.81096 |
| VAR00003 | 12 | 72.00 | 86.00 | 77.7500 | 4.26668 |
| VAR00004 | 12 | 68.00 | 85.00 | 77.5000 | 4.54273 |
| Valid N (listwise) | 12 |  |  |  |  |

C4 (17cases)

| case | gender | age | PTA (°) | | PLMA (°) | |
| --- | --- | --- | --- | --- | --- | --- |
|  |  |  | C4R | C4L | C4R | C4L |
| 1 | M | 47 | 44 | 48 | 74 | 81 |
| 2 | M | 48 | 42 | 44 | 83 | 79 |
| 3 | M | 50 | 43 | 51 | 79 | 72 |
| 4 | F | 57 | 38 | 41 | 79 | 93 |
| 5 | M | 40 | 47 | 49 | 81 | 83 |
| 6 | M | 42 | 42 | 43 | 89 | 78 |
| 7 | M | 44 | 47 | 42 | 79 | 77 |
| 8 | F | 61 | 38 | 41 | 77 | 76 |
| 9 | M | 49 | 46 | 39 | 76 | 78 |
| 10 | F | 48 | 47 | 41 | 78 | 74 |
| 11 | F | 61 | 45 | 42 | 78 | 75 |
| 12 | M | 52 | 48 | 41 | 76 | 80 |
| 13 | F | 64 | 41 | 45 | 74 | 81 |
| 14 | M | 52 | 45 | 49 | 83 | 79 |
| 15 | F | 60 | 43 | 40 | 79 | 72 |
| 16 | M | 70 | 41 | 42 | 79 | 93 |
| 17 | M | 52 | 37 | 42 | 81 | 83 |

| Descriptive Statistics | | | | | |
| --- | --- | --- | --- | --- | --- |
|  | N | Minimum | Maximum | Mean | Std. Deviation |
| VAR00001 | 17 | 37.00 | 48.00 | 43.1765 | 3.41386 |
| VAR00002 | 17 | 39.00 | 51.00 | 43.5294 | 3.59022 |
| VAR00003 | 17 | 74.00 | 89.00 | 79.1176 | 3.65517 |
| VAR00004 | 17 | 72.00 | 93.00 | 79.6471 | 6.02019 |
| Valid N (listwise) | 17 |  |  |  |  |

C5 (21 cases)

| case | gender | age | PTA (°) | | PLMA (°) | |
| --- | --- | --- | --- | --- | --- | --- |
|  |  |  | C5R | C5L | C5R | C5L |
| 1 | M | 47 | 48 | 44 | 76 | 85 |
| 2 | M | 48 | 39 | 44 | 83 | 86 |
| 3 | M | 50 | 45 | 42 | 84 | 78 |
| 4 | F | 57 | 33 | 40 | 83 | 81 |
| 5 | M | 40 | 42 | 42 | 94 | 96 |
| 6 | M | 42 | 41 | 49 | 85 | 78 |
| 7 | M | 44 | 40 | 44 | 86 | 87 |
| 8 | F | 61 | 43 | 47 | 78 | 88 |
| 9 | M | 49 | 41 | 42 | 81 | 90 |
| 10 | F | 48 | 46 | 44 | 77 | 79 |
| 11 | F | 61 | 43 | 39 | 82 | 80 |
| 12 | M | 52 | 42 | 43 | 84 | 83 |
| 13 | F | 64 | 41 | 40 | 87 | 79 |
| 14 | M | 52 | 51 | 48 | 88 | 80 |
| 15 | F | 60 | 40 | 42 | 90 | 83 |
| 16 | M | 70 | 41 | 40 | 79 | 85 |
| 17 | M | 52 | 44 | 42 | 84 | 81 |
| 18 | M | 58 | 49 | 46 | 83 | 79 |
| 19 | F | 56 | 39 | 38 | 85 | 81 |
| 20 | F | 27 | 42 | 41 | 81 | 79 |
| 21 | M | 59 | 38 | 38 | 79 | 80 |

| Descriptive Statistics | | | | | |
| --- | --- | --- | --- | --- | --- |
|  | N | Minimum | Maximum | Mean | Std. Deviation |
| VAR00001 | 21 | 33.00 | 51.00 | 42.2857 | 4.01426 |
| VAR00002 | 21 | 38.00 | 49.00 | 42.6190 | 3.07370 |
| VAR00003 | 21 | 76.00 | 94.00 | 83.2857 | 4.36054 |
| VAR00004 | 21 | 78.00 | 96.00 | 82.7619 | 4.62498 |
| Valid N (listwise) | 21 |  |  |  |  |

C6 (23 cases)

| case | gender | age | PTA (°) | | PLMA (°) | |
| --- | --- | --- | --- | --- | --- | --- |
|  |  |  | C6R | C6L | C6R | C6L |
| 1 | F | 52 | 44 | 36 | 95 | 91 |
| 2 | M | 57 | 39 | 40 | 88 | 85 |
| 3 | M | 75 | 38 | 37 | 82 | 84 |
| 4 | M | 48 | 39 | 40 | 82 | 83 |
| 5 | F | 47 | 40 | 36 | 89 | 92 |
| 6 | M | 48 | 35 | 44 | 78 | 84 |
| 7 | M | 50 | 44 | 43 | 94 | 89 |
| 8 | F | 57 | 31 | 39 | 101 | 102 |
| 9 | F | 58 | 44 | 45 | 90 | 83 |
| 10 | M | 56 | 37 | 44 | 98 | 92 |
| 11 | M | 27 | 35 | 35 | 82 | 86 |
| 12 | M | 59 | 38 | 39 | 84 | 82 |
| 13 | F | 50 | 36 | 32 | 82 | 84 |
| 14 | M | 44 | 45 | 40 | 90 | 86 |
| 15 | F | 46 | 32 | 34 | 81 | 86 |
| 16 | M | 43 | 34 | 39 | 93 | 82 |
| 17 | M | 50 | 44 | 36 | 91 | 91 |
| 18 | M | 58 | 43 | 40 | 86 | 88 |
| 19 | F | 47 | 45 | 42 | 80 | 82 |
| 20 | F | 62 | 35 | 38 | 91 | 83 |
| 21 | M | 68 | 34 | 29 | 92 | 90 |
| 22 | F | 63 | 37 | 36 | 83 | 86 |
| 23 | M | 46 | 41 | 47 | 82 | 83 |

| Descriptive Statistics | | | | | |
| --- | --- | --- | --- | --- | --- |
|  | N | Minimum | Maximum | Mean | Std. Deviation |
| VAR00001 | 23 | 31.00 | 45.00 | 38.6957 | 4.37384 |
| VAR00002 | 23 | 29.00 | 47.00 | 38.7391 | 4.30874 |
| VAR00003 | 23 | 78.00 | 101.00 | 87.5652 | 6.23645 |
| VAR00004 | 23 | 82.00 | 102.00 | 86.6957 | 4.73320 |
| Valid N (listwise) | 23 |  |  |  |  |

C7 (18cases)

| case | gender | age | PTA (°) | | PLMA (°) | |
| --- | --- | --- | --- | --- | --- | --- |
|  |  |  | C7R | C7L | C7R | C7L |
| 1 | F | 52 | 38 | 39 | 94 | 92 |
| 2 | M | 57 | 38 | 37 | 88 | 87 |
| 3 | M | 75 | 32 | 35 | 80 | 83 |
| 4 | M | 48 | 33 | 40 | 88 | 93 |
| 5 | F | 47 | 34 | 36 | 87 | 83 |
| 6 | M | 48 | 32 | 31 | 92 | 90 |
| 7 | M | 50 | 36 | 37 | 96 | 92 |
| 8 | F | 57 | 32 | 34 | 99 | 103 |
| 9 | M | 40 | 35 | 37 | 86 | 90 |
| 10 | F | 42 | 25 | 29 | 102 | 97 |
| 11 | M | 43 | 35 | 34 | 89 | 92 |
| 12 | M | 50 | 42 | 35 | 93 | 90 |
| 13 | M | 58 | 34 | 34 | 89 | 86 |
| 14 | F | 47 | 37 | 37 | 84 | 84 |
| 15 | F | 62 | 34 | 36 | 89 | 86 |
| 16 | M | 68 | 32 | 32 | 90 | 88 |
| 17 | F | 63 | 30 | 35 | 93 | 94 |
| 18 | M | 46 | 31 | 36 | 86 | 84 |

| Descriptive Statistics | | | | | |
| --- | --- | --- | --- | --- | --- |
|  | N | Minimum | Maximum | Mean | Std. Deviation |
| VAR00001 | 18 | 25.00 | 42.00 | 33.8889 | 3.70832 |
| VAR00002 | 18 | 29.00 | 40.00 | 35.2222 | 2.69106 |
| VAR00003 | 18 | 80.00 | 102.00 | 90.2778 | 5.34466 |
| VAR00004 | 18 | 83.00 | 103.00 | 89.6667 | 5.24685 |
| Valid N (listwise) | 18 |  |  |  |  |
